# Activation of Right Ventral Prefrontal Cortex Using a Predictive Cue during Visual Spatial Orienting of Attentional Processes: An fMRI Study

**DOI:** 10.1155/2012/961342

**Published:** 2012-05-07

**Authors:** Chunlin Li, Jinglong Wu

**Affiliations:** Biomedical Engineering Laboratory, Graduate School of Natural Science and Technology, Okayama University, Okayama 700-8530, Japan

## Abstract

Visual spatial orienting of attention can be investigated with location-cueing paradigms in which a cue provides correct information about the location of the upcoming target. Target detection is facilitated when the target appears at the expected cued location. In this study, we examined the brain activation of the spatial orienting response based on attentional “benefits.” During an fMRI experiment, two types of attentional tasks were used. Both predictive and nonpredictive cues were used and followed by an upcoming target. Behavioral data showed a faster reaction time with the predictive cue when compared with that of the nonpredictive cue. The fMRI results of these two tasks were compared, whereby isolated brain areas activated when the targets appeared at the attended position after a specific spatial expectation was induced by the cue were compared with when equivalent targets appeared after no spatial expectation was induced by the cue. The results showed that the right ventral prefrontal cortex was activated to a similar degree as the dorsal frontoparietal spatial attentional network.

## 1. Introduction

The visual spatial orienting of attention experimental paradigm is a widely studied model that was first developed by Posner [1] for studying the covert visual spatial orienting of attention. In this paradigm, an arrow can usually be used as a cue and presented in the center of the visual field. When a cue is presented, it points left or right in order to provide a spatial hint for the upcoming target, whereby the participants can predict the location of a target and pay attention to that location voluntarily. Functional magnetic resonance imaging (fMRI) studies have discovered a dorsal frontoparietal network, which includes the inferior parietal lobe (IPL) and the frontal eye field (FEF), and this network is involved in visual spatial orienting of attention [2–4]. In these goal-directed attention paradigms (spatial endogenous orienting of attention paradigm), a visual cue is usually presented centrally and provides a spatial prediction of the upcoming target [5]. When a cue provides the spatial hint correctly, it is called valid cue. When a cue provides the wrong spatial hint, it is called an invalid cue. The percentage of valid cue counts is called validity.

 By using a predictive cue with 100% validity [5], that is, a central visual spatial cue that indicates the location of an upcoming peripheral target, stimulus detection is facilitated when the target appears at the expected (i.e., the validly cued) location. The difference in reaction times between the neutral cued, which uses a nonpredictive cue, and the predictive cued target is referred to as the attentional “benefit” [6]. To investigate neural correlates of attentional benefits, Coull and Nobre [5] conducted an fMRI experiment and revealed that brain activation within the ventrolateral prefrontal cortex (VLPFC) activated the dorsal frontoparietal spatial attentional network. The VLPFC has been considered to serve the role of maintaining an endogenous alert state for target responses [7, 8] with right hemisphere dominance [9]. However, Rizzuto et al. used intracranial electroencephalogram (EEG) to investigate pointing movements [10], which suggested the involvement of the bilateral VLPFC (Brodmann area 45/47) in spatial selectivity and in the encoding of spatial information during both the target presence period and the pointing movement period.

 On the other hand, an fMRI study [6] showed that when the predictive cued condition used a cue validity of 80%, the threshold neuroimaging result within the VLPFC had no significant response. As mentioned in the above paragraph, Coull and Nobre [5] showed that the VLPFC was activated during the spatial attention orienting process. Although the Posner task was used in a similar manner, Doricchi et al. [6] showed that the activation within the VLPFC was absent. Namely, during visual spatial orienting of the attention process, the activation levels within the VLPFC were unclear when a different cue validity was used.

 To address this problem, we used a cue-target experimental paradigm. A cue-directed visual spatial orienting attention task using a visual predictive central cue (Directional task) was used to record the brain activation when participants shifted their attention under the cue information (cue-directed attention with 90% validity). Another visual spatial orienting of attention task using a visual neutral central cue (Nondirectional task) was used to record the activation involved with the nonpredictive cue. In a previous study, these earlier experiments may not have sufficiently controlled for activation from transient, detection-related processes evoked by hits [8]. Therefore, a control task was performed to exclude the brain activation resulting from pressing the response key. Our results showed that target responses based on predictive cues occurred within the right VLPFC and bilateral FEF, which is similar to what is observed in the bilateral IPL and superior parietal lobe. Our results support the conclusion of Coull and Nobre [5] that showed activation of the VLPFC during visual spatial orienting of attention.

## 2. Method and Materials

### 2.1. Participants and Tasks

The participants were 12 healthy right-handed male students aged 21–26 years with normal vision and hearing. Informed consent was obtained from each participant following a detailed explanation of the study. The study was approved by Kagawa University's Institutional Research Review Board.

**Figure 1:**
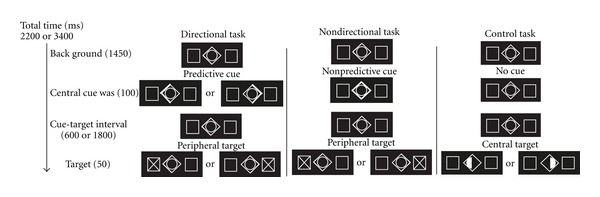
Experimental paradigm. In the Directional task, the visual cue indicates spatial information with a validity of 90%. The cue was illuminated for 100 ms and after the cue-target interval (600 or 1800 ms), the visual target was presented for 50 ms. During the Nondirectional task, a neutral cue was used that provided no spatial information. During the control task, the left or right half of the inner circle in the center of the screen would turn to white upon which the participants were asked press the reaction key. The reaction time was not recorded during this control task.

The three tasks used are shown in Figure 1. A predictive cue stimulus was used to direct the subjects' attention to one of two possible target locations (left or right) during the Directional task. Another cue stimulus, the nonpredictive cue, did not provide information during the Nondirectional task. The two cues were used separately for the Directional task and the Nondirectional task. For the Directional task, the predictive cue (90% validity) was presented at the center of the screen for 100 ms as an arrow with a diameter of two degrees of eccentricity. For the Nondirectional task, the entire central cue symbol was presented only for prompting the upcoming target. The third task was the control task, which was used as a baseline and could not be modeled with the regressors during data analysis. During the control task, either the right or left half of the central circle would turn white with an equal probability for each side. Participants were asked to press the appropriate response key when they detected a peripheral or central target and used the right index finger for the left target and the right middle finger for the right target. The control task was designed to account for any brain activation involved in pressing the appropriate response key. During the Directional and Nondirectional tasks, the interval length from the cue to the target was either 600 ms or 1800 ms with an equal probability for either delay. Participants were asked to gaze at the center of the cue and pay covert attention (without eye movement) to the target location. We recorded the reaction time as the length of time from the presentation of the visual target stimulus (“X” presented within the peripheral square with a width of two degrees of eccentricity, Figure 1) to the response given by the participant.

### 2.2. Procedures

Each participant performed one session, and the experimental details were explained to each subject before the MRI scan. A training course was also carried out before the scan. Participants were familiar with the task contents and eye fixation control. A block design was used in which ten trials of the same task were performed continuously and randomized between tasks with a summation of thirty trials for every task. The experiment lasted for a total of 270 seconds. Participants were told to press the key with high accuracy and as quickly as possible. While performing the experimental tasks, participants were required to pay attention to a specific type of cue (spatial or neutral). More specifically, participants were instructed to pay covert attention to a right or left field target based on the spatial cue in the Directional task. In the Nondirectional task, the cue only preceded the incoming target, and participants were told to anticipate that one of the two positions of the target would occur alternatively.

### 2.3. Functional MR Scanning

Images were acquired using a 1.5 T Philips scanner vision whole-body MRI system with a head coil to measure brain activation. The imaging area consisted of 32 functional gradient-echo planar imaging (EPI) axial slices (voxel size 3 × 3 × 4 mm, TR = 3000 ms, TE = 50 ms, FA = 90°, 64 × 64 matrix) that were used to obtain T2*-weighted fMRI images in the axial plane. For each task, we obtained 124 functional volumes and excluded the first 4 scans from analysis. Before the EPI scan, a T2-weighted volume was acquired for anatomical alignment (TR = 3500 ms, TE = 100 ms, FA = 90°, 256 × 256 matrix, voxel size = 0.75 × 0.75 × 4 mm). The T2 image acquisition used the same slices as the functional image acquisition.

### 2.4. Behavioral Data Analysis

Reaction times were used as behavioral data. The RT data during the fMRI experiment were analyzed by a paired *t*-test (SPSS 12.0j for Windows). For each task, 340 RT data points were acquired for the Directional and Nondirectional tasks, excluding trials where participants gave an incorrect response. A paired *t*-test was used to determine the statistical significance of the RT between the Directional and Nondirectional tasks. 

### 2.5. Imaging Data Analysis

We used MRIcro (http://www.mccauslandcenter.sc.edu/CRNL/) to change the Digital Imaging and COmmunication in Medicine (DICOM) files into MRIimg files and MRIhdr files. For each participant, the functional images of the first four volumes were excluded from the data analysis. The DICOM files from the 5th scan to the 124th scan were imported into MRIima as MRIhdr files. The DICOM files for the T2 images were also imported into MRIimg as MRIhdr files.

 Functional images were analyzed with statistical parametric mapping (SPM5, Wellcome Department of Cognitive Neurology, London, UK). The functional images from each task were realigned using the first scan as a reference. T2-weighted anatomical images were coregistered to the first scan in the functional images. Then, the coregistered T2-weighted anatomical images were normalized to standard T2 template images as defined by the Montreal Neurological Institute. Finally, these spatially normalized functional images were smoothed with an isotopic Gaussian kernel of 8 mm.

The statistical analysis was performed in two stages using a mixed-effects model. In the first-level analysis, neural activity was modeled by a *β* function at stimulus onset. These functions were then convolved with a canonical hemodynamic response function (HRF) and its temporal and dispersion derivatives [11] to yield regressors in a general linear model (GLM) that modeled the blood oxygen-level dependent (BOLD) response to each event type (Directional task versus Baseline and Nondirectional task versus Baseline). The time series in each voxel were high-pass filtered to 1/128 Hz to remove low-frequency noise and scaled within a session to a grand mean of 100 across both voxels and scans. Parameter estimates for events of interest were estimated using a general linear model. Nonsphericity of the error covariance was accommodated by an AR(1) (first-order autoregressive) model in which the temporal autocorrelation was estimated by pooling over suprathreshold voxels [12]. The parameters for each covariate and the hyperparameters governing the error covariance were estimated using ReML (restricted maximum likelihood).

 The “con” or contrast (difference in *β*) images of the first-level analysis were then used for the second-level group statistics (random effect analysis). To identify whole brain activation for the Directional task and Nondirectional tasks, a one-sample *t*-test analysis was performed. Only effects achieving an uncorrected threshold of *P* < 0.01 for multiple comparisons at the voxel level were interpreted for the Directional task and Nondirectional task. To further test the neural network for target responses based on visual spatial orienting of attention, a contrast map of the Directional task versus Nondirectional task was built with a threshold of an uncorrected *P* < 0.001 for multiple comparisons at the voxel level.

 For further test the neural correlations of spatial awareness based target response, we determined the anatomical regional mask for small-volume correction (SVC). Nine masks (Frontal_Mid_R for right dorsolateral prefrontal cortex; Frontal_Mid_Orb_R for right ventrolateral prefrontal cortex; Frontal_Mid_L for left dorsolateral prefrontal cortex; Frontal_Mid_Orb_L for left ventrolateral prefrontal cortex; R_Angular for right inferior parietal cortex; L_Angular for left inferior parietal cortex; Parietal_Sup_L for left superior parietal lobule; Parietal_Sup_R for right superior parietal lobule) were obtained from the automatic anatomy label (AAL), and a regional mask (bilateral Brodmann area 4/6) was obtained from TD Brodmann areas of WFU PickAtlas (http://fmri.wfubmc.edu/cms/software#PickAtlas). We used small-volume correction with a familywise error controlled at *P* < 0.05 for multiple comparisons at the voxel level for the analysis of Directional versus Nondirectional tasks.

## 3. Results

### 3.1. Behavioral Results

Behavioral data were derived from the participants' performance during the fMRI experiment. All participants responded to the tasks with accuracy above 90%. The reaction time (RT) was 323 ms (SD = 48.9) for the Directional task and 384 ms (SD = 53.5) for the Nondirectional task. A paired *t*-test with student adjustment showed a significant difference between the two tasks (*P* < 0.001). 

### 3.2. Functional Results

**Figure 2:**
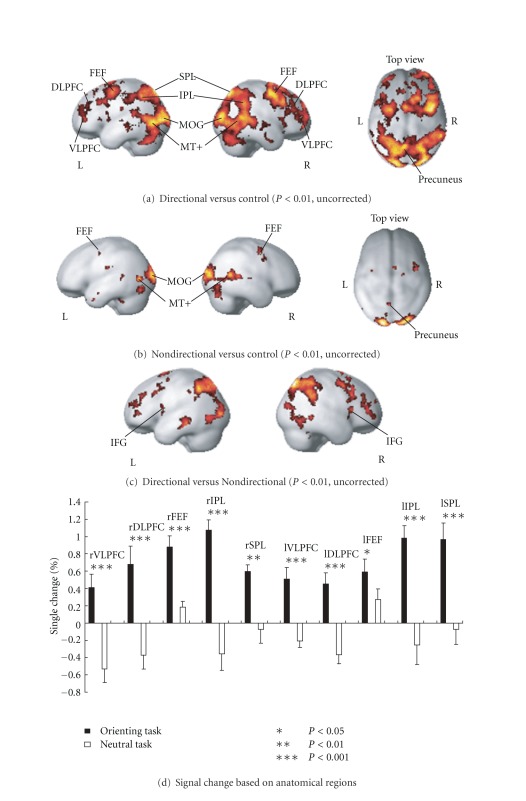
SPM results of (a) Directional task (*P* < 0.01, uncorrected, cluster size > 0 voxels) and (b) Nondirectional task (*P* < 0.01, uncorrected, cluster size > 0 voxels). A bilateral frontoparietal network is shown as a result of the Directional task, which is the same as the VLPFC. (c) Brain activation obtained by contrasting the Directional task and the Nondirectional task. (d) Regional BOLD signal change based on anatomical masks. L/l: left hemisphere, R/r: right hemisphere, SPL: superior parietal lobule, IPL: inferior parietal lobule, FEF: frontal eye field; DLPFC: dorsolateral prefrontal cortex, VLPFC: ventrolateral prefrontal cortex, MOG: middle occipital gyrus, MT+: middle temporal and superior temporal cortex, IFG: inferior frontal gyrus.

Figure 2 shows imaging results during the Directional task (Figure 2(a)), the Nondirectional task versus the control task (Figure 2(b)), and the contrast result of the Directional task versus the Nondirectional task (Figure 2(c)). We noted that bilateral superior parietal lobe (SPL, BA7) and IPL (BA40) were activated at a similar level as the bilateral frontal cortex, including the FEF, dorsolateral prefrontal cortex (DLPFC), and VLPFC during the Directional task. We were able to confirm the dorsal frontoparietal attention network only from the Directional task. Activation within the visual cortex that included the middle occipital gyrus (MOG) and medial temporal/medial superior temporal cortex (MT+) is considered to serve a role in basic visual cognition and motion cognition [13] for both Directional and Nondirectional tasks. Bilateral activation within the parietal cortex, specifically the SPL (BA7), which plays a role in spatial cognition [14–17], was associated with both Directional and Nondirectional tasks. The observed activation of the precuneus (BA5/7) has been associated with an attention shift spatially from left and right [18, 19]. The left prefrontal cortex was also activated but to a lesser degree compared with the right hemisphere. To confirm these results, SVC was performed for correcting the contrast result of the Directional task versus the Nondirectional task (summarized in Table 1), which indicated that the bilateral SPL, IPS, FEF, right DLPFC, and right VLPFC were significantly activated.

## 4. Discussion

### 4.1. Neural Correlates to Visual Spatial Orienting of Attention

Although this study used a blocked design and did not separate the target and cue stimuli, we were able to differentiate activation on the basis of the directional spatial orienting of attention from activity caused by nondirectional orienting of attention and by comparing data from the Directional task and Nondirectional task. Furthermore, because the control task was used to exclude activation due to button pressing by the right index and middle finger, we considered that the activation shown in Figure 1 was successfully disassociated from motor action. Our results (Figure 2(c) and Table 1) show widespread activation that includes the bilateral IPL and FEF, and these results are consistent with data from previous voluntary visual spatial attention studies [2–5].

 The SPL cortex usually activates during visual spatial orienting of attention processes [20, 21]. This region, which showed a significant change in activation during the Directional task, has an incontestably well-described role in spatial orienting cognition [22, 23]. The precuneus (BA5/7) has functions in shifting attention between the apparent position of target stimuli defined in the left and right hemifield [5, 18, 19]. Working memory within the DLPFC contributes to executive functioning and the storage of information related to events [2, 4, 24, 25]. In the present study, we found that rDLPFC activation was associated with spatial orienting of attention (see SVC results for rDLPFC). The task set used in this study aimed to manipulate spatial attention to a given target. Therefore, the rDLPFC was activated as a result of its considerable role in storing information about the task and related information [26]. In contrast, the Nondirectional task provided no clue about the target nor did it provide any particular rule to be followed.

### 4.2. Contribution of Right VLPFC with a Valid Cue for Target Response

 The right VLPFC is our main focus for target detection based on visual spatial orienting of attention. The conflicting results from previous studies may have been caused by different responses within the right VLPFC, which includes detection-related processes evoked by hits [8] or target-related responses based on spatial orienting of attention [5]. We considered that our results may be caused by the latter because the detection-related processes evoked by these hits were excluded by a control task.

 From the imaging results of the Directional task and Nondirectional task (Figures 2(a) and 2(b)), we first confirmed that the activation within the right VLPFC only occurred during the Directional task. Furthermore, the contrast result (Figure 2(c) and Table 1), which was built to confirm the spatial orienting of attentional benefits for a target response, also showed activation of the right VLPFC to a similar degree as the frontoparietal spatial attentional network. This result is consistent with several visual spatial orienting of attention studies [5, 9] when cue validity was 100%. In contrast, an event-related study that used a low cue validity of 65% (averaged by 80% and 50%) [6] compared the valid condition to the neutral condition (similar to current study) and did not find activation within the right VLPFC. Accordingly, we considered that the right VLPFC was correlated with the spatial orienting response under highly spatial awareness, which is consistent with its role in directional maintenance of an alert state [7, 8].

 In addition, we did not consider that the right VLPFC was activated by a false alarm with the 10% invalid cue rate, which is because the false alarm response usually activates the inferior frontal gyrus (IFG) [7] (showed in Figure 2(c) of this study). We also did not consider that activation within the right VLPFC was caused by a cue stimulus because the changed activation by cue validity cognition itself usually occurs within the parietal area and FEF [27]. For the hemispheric asymmetries, Shulman et al. [9] noted that the widespread hemispheric asymmetries observed during target detection may partly reflect the involvement of diffusely projecting neuromodulatory systems, such as the locus coeruleus/noradrenaline (LC/NE) system, which has long been linked to alerting stimuli and arousal [28–30]. Furthermore, the LC/NE system has been proposed to facilitate transitions between behavioral states, including those related to shifts of attention and target detection [31]. Accordingly, the LC/NE system may have modulated the activity evoked in the widely distributed right hemisphere regions (which was distinct within the DLPFC and VLPFC as shown in Table 1) during the transition from monitoring an attended hemifield to a target detection/response.

In summary, we measured brain activity by fMRI both in a visual spatial orienting of attention task and a neutral visual directional task. We found a correlation within the VLPFC to a target response based on directional visual spatial orienting of attention. We conclude that, during a target response based on top-down attention processing, while the bilateral dorsal frontoparietal neural network was needed, the bilateral SPL and right VLPFC contributed to endogenous spatial orienting based on a valid cue.

## Figures and Tables

**Table 1:** Small volume correction results based on contrast of the Directional task versus the Nondirectional task. The *x*, *y*, and *z* coordinates are from SPM5. SPL: superior parietal lobe, IPL: inferior parietal lobe, FEF: frontal eye field, DLPFC: dorsolateral prefrontal cortex; VLPFC: ventrolateral prefrontal cortex, R: right hemisphere, L: left hemisphere, M: bilateral hemisphere.

	Regions	MNI coordinates			*P* value (familywise error)
		*x*	*y*	*z*	
1	Right VLPFC	38	58	−4	<0.05
		42	54	−4	<0.05
2	Right DLPFC	46	38	18	=0.051
3	Right FEF	12	16	62	<0.02
		10	12	64	<0.02
4	Right SPL	24	−74	56	<0.001
		38	−68	52	<0.02
		40	−46	60	<0.02
		36	−48	56	<0.05
		32	−56	58	<0.05
5	Right IPL	58	−46	38	<0.001
		28	−74	48	<0.001
		44	−62	52	<0.02
		42	−46	60	<0.02
		38	−48	44	<0.02
		44	−46	54	<0.03
		40	−48	56	<0.03
		40	−64	52	<0.03
		56	−34	50	<0.03
		46	−58	52	<0.03
		44	−50	42	<0.04
		48	−52	42	<0.04
		36	−48	54	<0.05
6	Left VLPFC				ns
7	Left DLPFC				ns
8	Left FEF	−4	4	72	<0.005
		−4	4	66	<0.04
9	Left SPL	−42	−52	56	<0.005
		−40	−56	56	<0.005
		−14	−78	54	<0.02
		−32	−70	48	<0.02
		−28	−72	48	<0.02
		−18	−76	52	<0.02
		−22	−72	50	<0.02
		−34	−64	50	<0.03
		−22	−78	48	<0.05
		−36	−60	50	<0.05
10	Left IPL	−48	−52	44	<0.001
		−44	−52	54	<0.002
		−36	−52	46	<0.02
		−34	−56	52	=0.054
